# Characterization of the Binding Properties of Ten Aptamers Using the Intrinsic Fluorescence of Oxytetracycline

**DOI:** 10.1002/open.202300250

**Published:** 2024-02-05

**Authors:** Yichen Zhao, Biwen Gao, Juewen Liu

**Affiliations:** ^1^ Department of Chemistry Waterloo Institute for Nanotechnology University of Waterloo Waterloo Ontario N2L 3G1 Canada

**Keywords:** tetracycline, aptamers, fluorescence, biosensors, antibiotics

## Abstract

Tetracyclines are a class of commonly used four‐ringed antibiotics. A series of DNA aptamers were recently obtained using the capture‐SELEX (systematic evolution of ligands by exponential enrichment) method to bind to oxytetracycline, and one of the aptamers can bind to a few other tetracycline antibiotics as well. Upon binding to the aptamers, the intrinsic fluorescence of tetracycline antibiotics can be enhanced. At least 10 different DNA aptamers were isolated from the previous selection experiment. In this work, a systematic characterization of these ten aptamers was performed. Each of these aptamers shows a different degree of fluorescence enhancement ranging from around 1‐fold to over 20‐fold. Fluorescence enhancement was boosted in the presence of Mg^2+^. Isothermal titration calorimetry (ITC) studies were done and showed a great variety in dissociation constant (*K*_d_) from 62 nM to 1.6 μM. Steady‐state fluorescence spectroscopy and fluorescence lifetime studies showed a correlation between fluorescence lifetime and degree of fluorescence enhancement. A few aptamers showed slow binding kinetics, although no correlation was found between the kinetics of fluorescence change and degree of fluorescence enhancement. This is the first study of ten different aptamers for the same target, providing fundamental insights into aptamer binding and bioanalytical applications.

## Graphical abstract

Taking advantage of the intrinsic fluorescence of oxytetracycline, its binding to ten different DNA aptamers was characterized using fluorescence quantum yield, binding kinetics, fluorescence lifetime and calorimetry, offering insights into aptamer binding to small molecules.

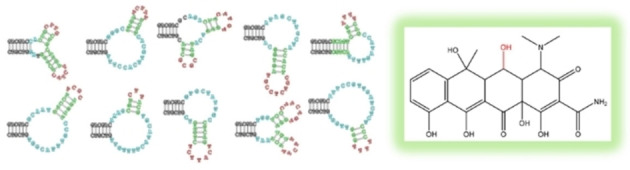

## Introduction

Tetracyclines cover a large family of over ten antibiotics with a common four‐ring core structure. The early ones were discovered in the 1940s, such as oxytetracycline (Terramycin) and chlortetracycline (Aureomycin). They are effective antibiotics inhibiting bacterial growth by impeding aminoacyl‐tRNA attachment and bacterial protein synthesis.^[1]^ However, it was soon discovered that these tetracyclines have serious side effects such as staining of the bones and teeth and are nowadays only used in animals. Some newer tetracyclines such as doxycycline were found to be more stable and less toxic to humans and remained used to date.

There has been a lot of interest in detecting tetracycline antibiotics.[^2^, ^3^, ^4^] Aptamers are single‐stranded DNA or RNA oligonucleotides that can selectively bind to target molecules.[^5^, ^6^, ^7^] Since the action of tetracyclines is to interact with RNA, development of aptamer‐based sensors has been attempted for a long time. An RNA aptamer for tetracycline was selected in 2001 by Berens et al by immobilization of tetracycline on sepharose beads.^[8]^ A few DNA aptamers were also reported by the Gu group using a similar target immobilization method.[^9^, ^10^]

We recently reported a number of DNA aptamers for tetracycline antibiotics that could all enhance the intrinsic fluorescence of the antibiotics.[^11^, ^12^] This fluorescence property allowed us to explore a new method for their detection and for fundamental binding assays.[^13^, ^14^, ^15^, ^16^] Only the fluorescence of tetracyclines could be enhanced by adding the aptamers. The fluorescence intensity of other molecules, regardless of whether they fluoresce or not, would not the changed by the addition of the aptamers.^[12]^

Due to the relatively large structure of tetracyclines, there are many ways for aptamers to bind. We observed at least ten families of DNA aptamer sequences.^[11]^ To better understand the binding of aptamers to tetracyclines, it is important to perform systematic binding assays. The intrinsic fluorescence of tetracyclines allows various ways to study binding. More importantly, we want to understand why some aptamers enhance the fluorescence more than others. Since oxytetracycline (OTC) was used as a target to obtain the aptamers, in this study, it was used as the ligand for the binding assays.

## Materials and Methods

### Chemicals

All of the DNA samples were purchased from Integrated DNA Technologies (Coralville, IA, USA). OTC and other chemicals were from Sigma‐Aldrich. Milli‐Q water was used to prepare all the buffers and solutions. Solutions of 100 nM OTC were diluted in a buffer of 10 mM MES pH 6.0, 50 mM NaCl, and 2 mM MgCl_2_. Each day, OTC solutions were prepared fresh by weighing new powders.

### Fluorescence Spectroscopy

The experiments were performed on a Tecan Spark microplate reader with an excitation wavelength set at 370 nm and an emission at 535 nm. For titrations, 100 nM OTC was dissolved in buffer (10 mM MES, pH 6.0, 50 mM NaCl, 0 or 2 mM MgCl_2_). The 10 aptamers were titrated such that the final volume change was kept to be less than 10 %. The solution was well mixed after each titration and allowed to equilibrate for 1 min before reading. To study the effect of Mg^2+^, 100 nM OTC was dissolved in buffer (10 mM MES, pH 6.0, 50 mM NaCl, 0, 0.2, 1, 2 and 5 mM MgCl_2_). The OTC2 aptamers were titrated.

### Kinetics of OTC binding

The experiments were performed on a Varian Eclipse fluorimeter with the excitation wavelength set for OTC at 370 nm while the emission wavelength was set to 530 nm (slit was 20 nm, high voltage mode). To determine the actual kinetic of aptamers binding to OTC, 100 nM OTC was dissolved in 500 μL buffer (10 mM MES, pH 6.0, 50 mM NaCl and 2 mM MgCl_2_), and its background fluorescence was monitored. After 1 min, each of the 10 aptamers was added to the solution, and the kinetics were monitored every 10 sec. This process was run in triplicate. To monitor the temperature dependence of aptamer bound OTC, temperatures were set to 12 °C, 22 °C and 32 °C. After the temperature reached each targeted value, background fluorescence was monitored for 100 nM OTC in 500 μL buffer (10 mM MES, pH 6.0, 50 mM NaCl and 2 mM MgCl_2_). After 1 min, the OTC43 or OTC2 aptamer was added to the solution, and the kinetics were monitored every 10 sec. This experiment was run in triplicate.

### Fluorescence Lifetime

To measure the lifetime of free OTC and the aptamer bound OTC, the experiments were performed on a Horiba Fluorimeter. A solution of 1 μM OTC was dissolved in 500 μL buffer (10 mM MES, pH 6.0, 50 mM NaCl and 2 mM MgCl_2_). In addition, samples were prepared to contain 2 μM of aptamers. The excitation was a laser with a wavelength of 450 nm, and the emission monochromator was set at 520 nm. The detector voltage ranged from 750 volts to 900 volts, and the measurement range was set to 100 ns and the data were collected for 10000 counts. Each experiment was performed in triplicate.

### Isothermal Titration Calorimetry (ITC)

Isothermal titration calorimetry was performed using a MicroCal VP‐ITC as described previously.[^12^, ^17^] 150 μM of OTC was titrated into 9 μM of aptamer. The aptamer and target were both dissolved in pH 6.0 MES buffer with 50 mM NaCl and 2 mM MgCl_2_. The background heat of target into plain buffer was subtracted and the data was analyzed using the accompanying Origin software.

## Results and Discussion

### The ten oxytetracycline binding aptamers

**Figure 1:**
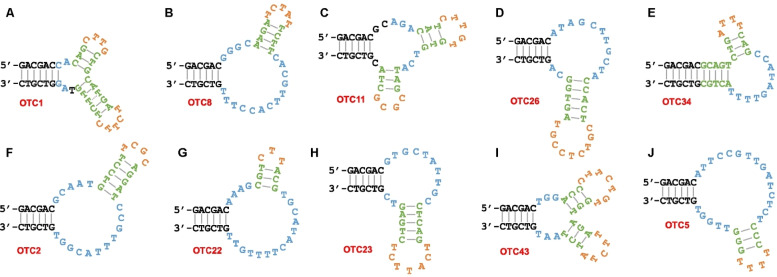
The Mfold predicted secondary structures of the ten OTC aptamers studied in this work: (A) OTC1, (B) OTC8, (C) OTC11, (D) OTC26, (E) OTC34, (F) OTC2, (G) OTC22, (H) OTC23, (I) OTC43 and (J) OTC5. Figure adapted from Ref.^[11]^ with permission. Copyright Royal Society of Chemistry 2023.

The secondary structures of the ten aptamers studied in this work are shown in Figure 1.^[11]^ They all have the common structure of at least six base pairs in the ends. In addition, each aptamer has at least one internal stem‐loop structure while some have two. The nucleotides marked in blue are conserved regions based on sequence alignment. Typically internal hairpins are not involved in target binding and binding likely takes place in the loops marked in blue. Upon binding to tetracycline antibiotics, they showed various extents of fluorescence enhancement from around 1‐fold to over 20‐fold.^[11]^ The presence of so many different aptamers for OTC could be related to the relatively large structure of OTC, allowing different ways of aptamer binding. The following studies aims to understand why the fluorescence enhancement of the aptamers are so diverse as well as more fundamental studies of the binding reactions.

### Effect of Mg^2+^

**Figure 2:**
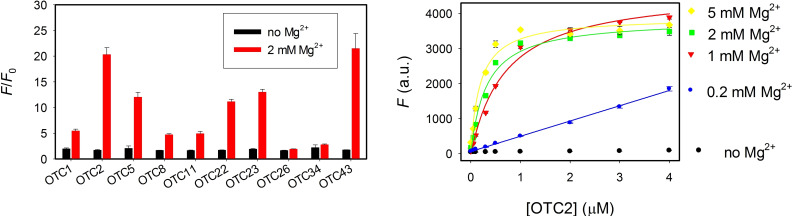
(A) The fluorescence intensity ratios of 100 nM OTC in the presence and absence of 2 μM of various aptamers. (B) The fluorescence of 100 nM OTC in the presence various concentrations of OTC2 aptamer at different Mg^2+^ concentrations.

We first studied the effect of Mg^2+^ on aptamer binding, which was able to affect the intrinsic fluorescence of OTC,^[18]^ and also the binding reaction.^[15]^ We added 2 μM aptamer to 100 nM OTC and compared the fold of fluorescence increase brought upon by the aptamers (*F*/*F_0_*, Figure 2A). Without Mg^2+^, the fluorescence enhancement of OTC due to aptamer binding was less than 100 % for all the ten aptamers (black bars), and all the aptamers behaved similarly. With 2 mM Mg^2+^, the fluorescence enhancement was quite diverse. Except for OTC26 and OTC34, which showed little fluorescence change even with Mg^2+^, the fluorescence increase of the other eight aptamers was significantly enhanced by Mg^2+^. In general, Mg^2+^ can promote the intrinsic fluorescence of OTC, but with Mg^2+^, the fluorescence with aptamer was even higher.^[15]^ Since OTC2 showed a large fluorescence enhancement, we also measured the aptamer binding in the presence of various concentrations of the aptamer (Figure 2B). Without Mg^2+^, no binding occurred regardless of the OTC2 aptamer concentration. Even concentrations as low as 0.2 mM Mg^2+^ promoted binding. The effect of Mg^2+^ binding saturated around 2 mM Mg^2+^. Therefore, in the subsequent studies, we included 2 mM Mg^2+^ for all the experiments.

### OTC binding kinetics

**Figure 3:**
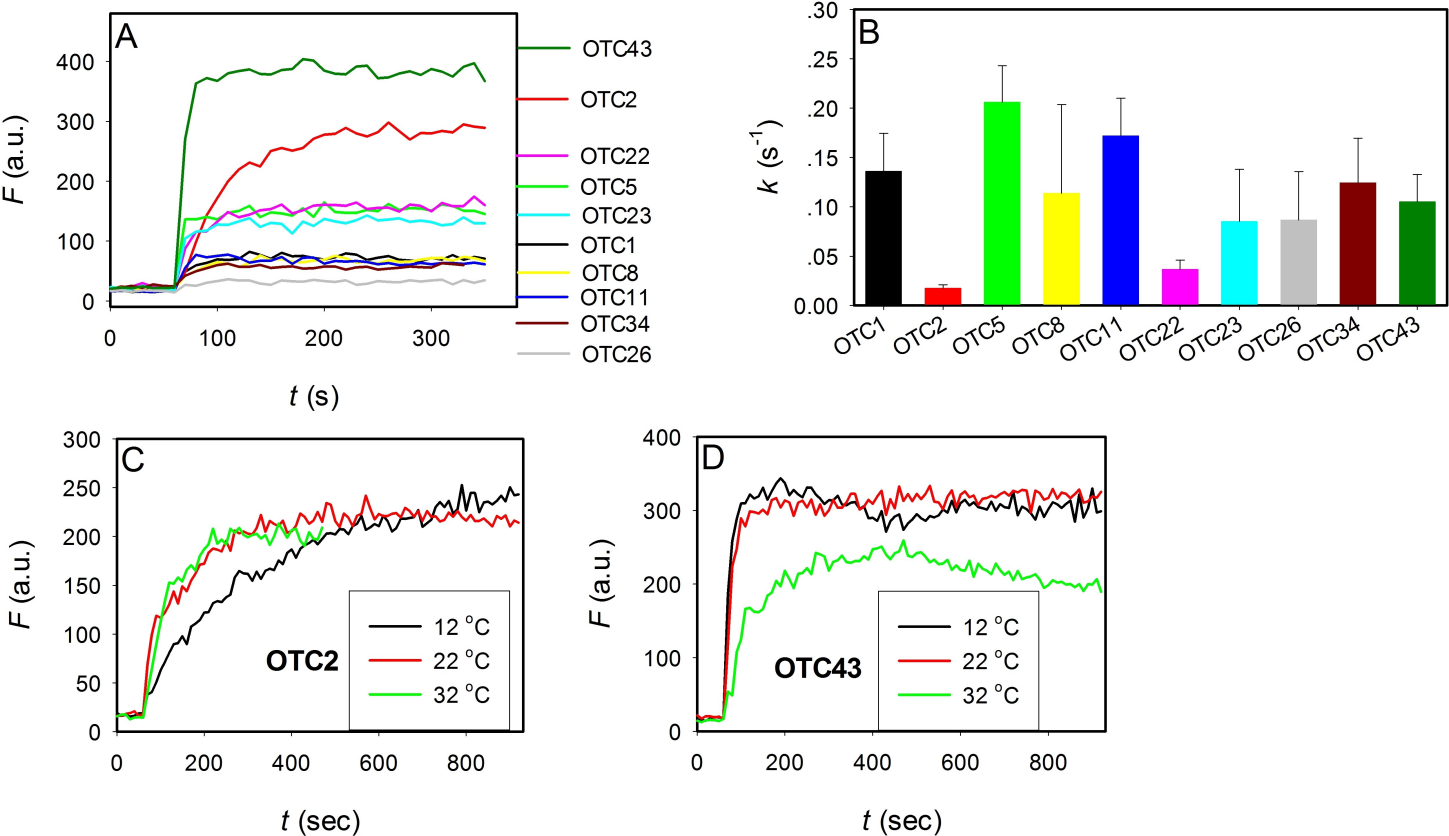
(A) Kinetics of fluorescence enhancement upon 2 μM aptamer binding to 100 nM OTC5. (B) The first‐order rate constant of these aptamers based on the data in (A). Temperature‐dependent binding kinetics of (C) OTC2 aptamer and (D) OTC43 aptamers.

**Figure 4:**
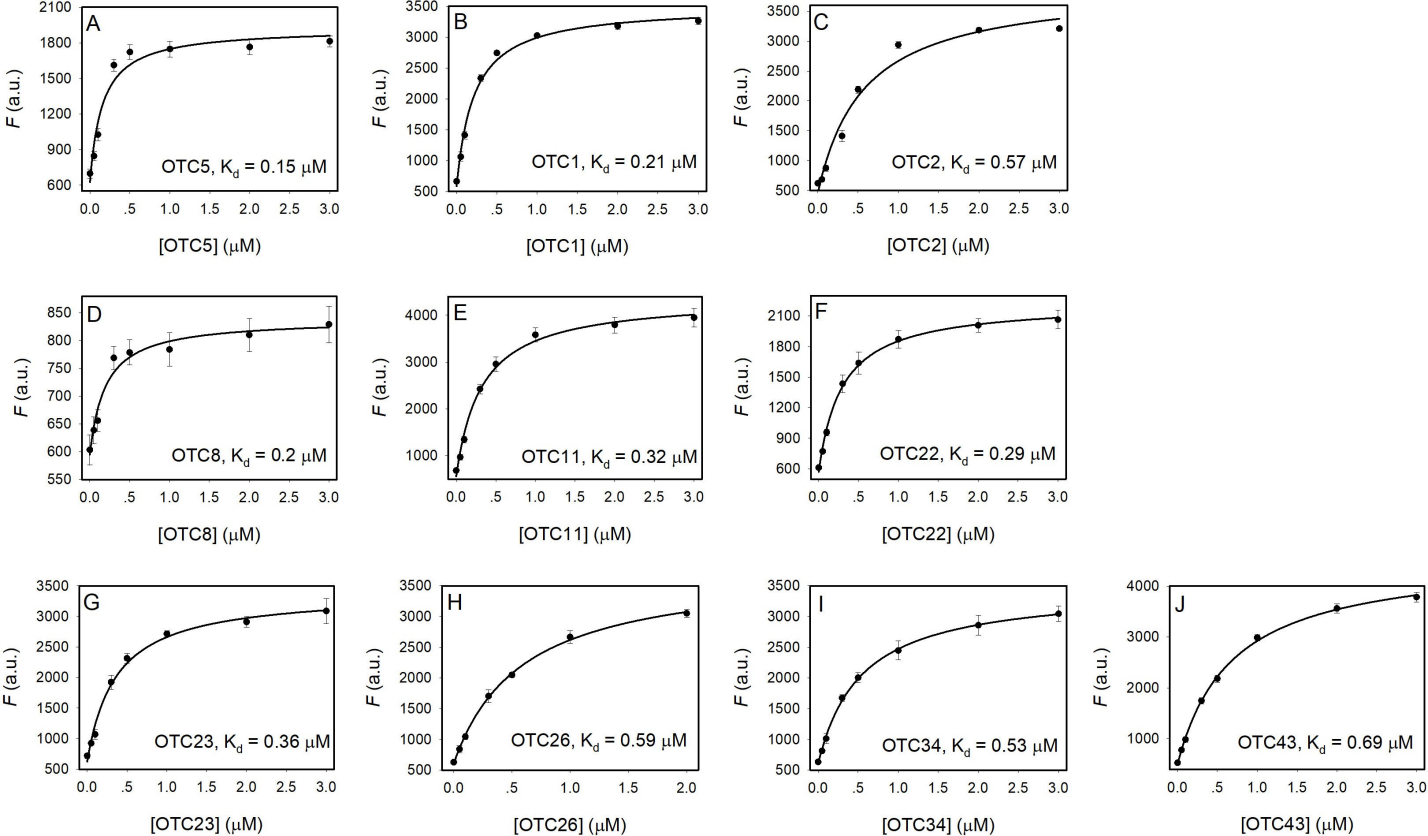
Titration curves of various aptamers: (A) OTC5, (B) OTC1, (C) OTC2, (D) OTC8, (E) OTC11, (F) OTC22, (G) OTC23, (H) OTC26, (I) OTC34 and (J) OTC43.

Aptamer binding to small molecules usually happen quite fast, although little binding kinetic information is available.^[19]^ The intrinsic fluorescence of OTC allowed a convenient way to characterize binding kinetics. We monitored the fluorescence of 100 nM OTC for 1 min and then added 2 μM of an aptamer and the kinetics was continuously monitored (Figure 3A). We can see a separation in the fluorescence increase kinetics into two groups. We plotted the fitted first‐order rate constants, and OTC2 followed by OTC22 were the obvious slower ones, while the rest all showed a similarly fast rate of binding with a rate constant around 0.1 s^−1^ (Figure 4B). Note that this is only an estimation and we cannot measure such fast kinetics using manual operations.

There was however no clear correlation between the amount of fluorescence enhancement and the kinetics of fluorescence change. For example, OTC43 and OTC2 showed the highest fluorescence increase but their rates were quite different. The slowest OTC2 reached saturated binding within 3 min after mixing. It might be that the binding of the OTC2 aptamer to OTC requires a conformational change, which needs energy. To confirm this hypothesis, we then did the kinetics with OTC2 and OTC43 at different temperatures (Figure 4C, 4D). Interestingly, OTC2 showed faster binding at higher temperatures, while OTC43 showed slower binding at higher temperatures. We reason that OTC43 does not have an energy barrier related to aptamer conformational change even at 12 °C. At higher temperature, binding reactions are disfavored due to higher entropy contributions. For OTC2, while the binding state is thermodynamically more stable, a kinetic barrier associated with aptamer conformational change existed at 12 °C. Even at 32 °C, the barrier is still partially there to show a slower binding kinetics.

By examining the secondary structure of OTC2 and OTC22, they showed some similarities. It might be that they can form some internal secondary and even tertiary structures. For example, OTC22 may form base pairs in the blue regions. On the other hand, some faster binding ones such as OTC5 also showed a similar secondary structure. More detailed understanding might be achieved via further structural biology studies such as NMR spectroscopy. We reason that the slower binding ones might be useful for studying aptamer binding, while the faster binding ones are more useful as biosensors to achieve a quicker sensor response.

### High affinity binding aptamers

We then measured the binding affinities of these aptamers by gradually titrating each aptamer into 100 nM of OTC solution. For all the aptamers, they induced gradually enhanced fluorescence. Note that each figure has a different *y*‐axis scale to best show the binding curves. We previously reported the *K*_d_ of OTC5 aptamer to be 0.15 μM (Figure 4A). All the other aptamers showed nanomolar binding affinities. At this point, it is unclear whether all the aptamers bind OTC in different ways or whether some aptamers belong to the same family. Nevertheless, we have provided a large group of different aptamers for tetracycline antibiotics.

### Fluorescence lifetime

**Figure 5:**
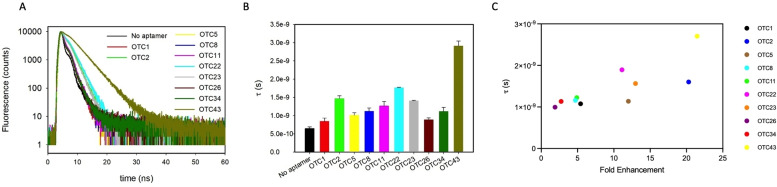
(A) Fluorescence lifetime decay traces of 1 μM OTC alone and in the presence of 2 μM aptamers. (B) Average lifetime of the samples. (C) Average fluorescence lifetime versus the fold of fluorescence enhancement. The measurements were performed in 10 mM pH 6.0 MES buffer with 50 mM NaCl and 2 mM MgCl_2_.

To further understand the mechanism of fluorescence enhancement, fluorescence lifetime measurements were performed. Free OTC has a short sub‐nanosecond lifetime in aqueous solutions,^[20]^ and our 1 μM OTC showed an average lifetime of 0.65 ns. When 2 μM aptamers were added, the fluorescence lifetime all increased and the largest increase was seen for the OTC43 aptamer, which also had the highest fluorescence intensity enhancement (Figure 5A, 5B). When we plotted the average lifetime of OTC in the presence of each aptamer, a good correlation between fluorescence intensity increase and lifetime can be observed (Figure 5C). Fluorescence quantum yield can be written as Φ=τ/τ_n_, where τ and τ_n_ are the measured lifetime and intrinsic lifetime (when quantum yield is 1). Therefore, the increased OTC quantum yield in the presence of the aptamer can be explained by the increased lifetime (Figure 5C). We reason that the aptamers provide a more hydrophobic environment for OTC, which decreased collisions with water molecules and thus decreased the rate of non‐radiative decay.

### Isothermal Titration Calorimetry

**Figure 6:**
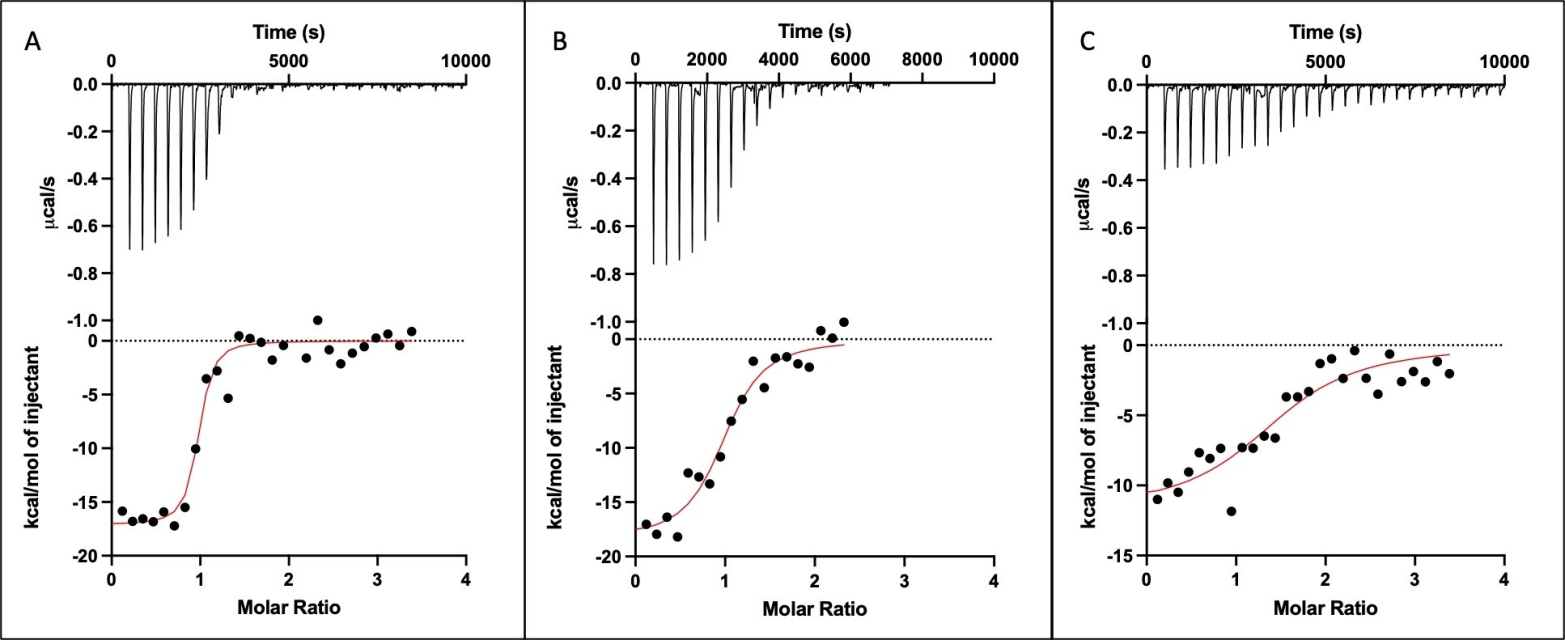
ITC traces of (A) OTC2, (B) OTC22, and (C) OTC43. All experiments were done in 10 mM pH 6.0 MES buffer with 50 mM NaCl and 2 mM Mg^2+^, 9 μM aptamer and 150 μM OTC at 25 °C.

To further understand aptamer binding, we picked a few of them for isothermal titration calorimetry (ITC) study (Figure 6). We picked OTC2 and OTC22 for their slow kinetics. We also picked OTC43 for its very large fluorescence enhancement. All the binding reactions were exothermic. Both OTC2 and OTC22 showed a similar 1 : 1 binding of aptamer to target molecule, although OTC2 had a *K*_d_ that was ~8 times lower than OTC22. OTC43 had an even higher *K*_d_ of >1 μM. OTC43 ((Figure 6C) also generated less heat than OTC2 or OTC22 which were similar to each other. Both the enthalpy and entropy values for OTC2 and OTC22 were similar but OTC43 had a smaller Δ*H* and less negative Δ*S*.

From the fluorescence titration in Figure 4C, the OTC2 aptamer has a *K_d_* of 0.57 μM when reading afer 1 minute of equilibration. This *K*_d_ was 9‐fold higher than that from ITC. Since OTC2 has slower kinetics than the other aptamers and takes around 10 min to fully react with the OTC (Figure 3C), another fluorescence assay was run with OTC2 after 12 min of equilibration (Figure S1). A *K_d_* of 0.081 μM was obtained in this case, which is closer to the ITC value of 0.062 μM (Table 1). Overall, the *K*_d_ values obtained from ITC and the fluorescence titration were quite consistent with each other.

**Table 1:** Fitted thermodynamic values of ITC traces.

**Aptamer**	**Target**	***N***	***K***_**d**_ **(μM)**	**Δ*H* (cal/mol) (x10^4^)**	**Δ*S* (cal/mol/K)**
**OTC2**	OTC	0.93±0.02	0.062±0.02	−1.7±0.7	−24.1
**OTC22**	OTC	0.99±0.05	0.49±0.11	−1.8±0.1	−32.9
**OTC43**	OTC	1.5±0.1	1.6±0.57	−1.1±0.1	−12.9

Previous work has also reported RNA aptamers that bind tetracyclines and enhance their fluorescence. Some RNA aptamers that were isolated have *K*_d_ from 1.7 nM to 600 nM for binding with OTC.^[16]^ Another group reported RNA aptamers with *K*_d_ of 7.6 nM that were specific for an analogue of OTC, doxycycline.^[21]^ These *K*_d_ values were also determined using fluorescence titration spectroscopy and ITC. The RNA aptamers could enhance the intrinsic fluorescence of the tetracycline antibiotics around 2–3 fold with the doxycycline RNA aptamer able to reach around 6‐fold enhancement. Doxycycline is known to have better fluorescence enhancement than that OTC or tetracycline upon aptamer binding^[11]^ and has also been shown with these DNA aptamers. Overall, the intrinsic fluorescence of tetracycline antibiotics is a great way to characterize aptamer binding.

## Conclusions

The intrinsic fluorescence of OTC allowed us to conveniently characterize its binding by aptamers. In this work we described ten aptamers for oxytetracycline that enhance its intrinsic fluorescence to various degrees. Fluorescence enhancement was probed using fluorescence titration spectroscopy and isothermal titration calorimetry. This is the first study in the aptamer field to characterize up to ten aptamers to the same target. Most targets have just one or two representative aptamers. The tetracyclines have a larger structure allow different ways of target binding by aptamers. These ten aptamers all had high binding affinities with nine of them showing nanomolar affinities. They all require Mg^2+^ to promote binding. The increased fluorescence intensities correlated with the increased fluorescence lifetimes and some aptamers showed different reaction kinetics. These aptamers can be easily adapted to different applications. For example, combining different aptamers, more selective detection of OTC can be achieved. In addition, faster binding aptamers can be used for biosensing while slower aptamers can be used to further probe the binding mechanism of the aptamer and its target molecule.

## Conflict of interests

The authors declare no conflict of interest.

1

## Supporting information

As a service to our authors and readers, this journal provides supporting information supplied by the authors. Such materials are peer reviewed and may be re‐organized for online delivery, but are not copy‐edited or typeset. Technical support issues arising from supporting information (other than missing files) should be addressed to the authors.

Supporting Information

## Data Availability

The data that support the findings of this study are available from the corresponding author upon reasonable request.
