# Supplementary material for: Characterization of the Binding Properties of Ten Aptamers Using the Intrinsic Fluorescence of Oxytetracycline
Source: ChemistryOpen. 2024 Feb 5;13(7):e202300250. doi: 10.1002/open.202300250 (PMC11230938; doi:10.1002/open.202300250)
Supplement: Supplementary file 1 — Supporting Information [file OPEN-13-e202300250-s001.pdf]

ChemistryOpen

Supporting Information

Characterization of the Binding Properties of Ten Aptamers Using the Intrinsic Fluorescence of Oxytetracycline

Yichen Zhao, Biwen Gao, and Juewen Liu*

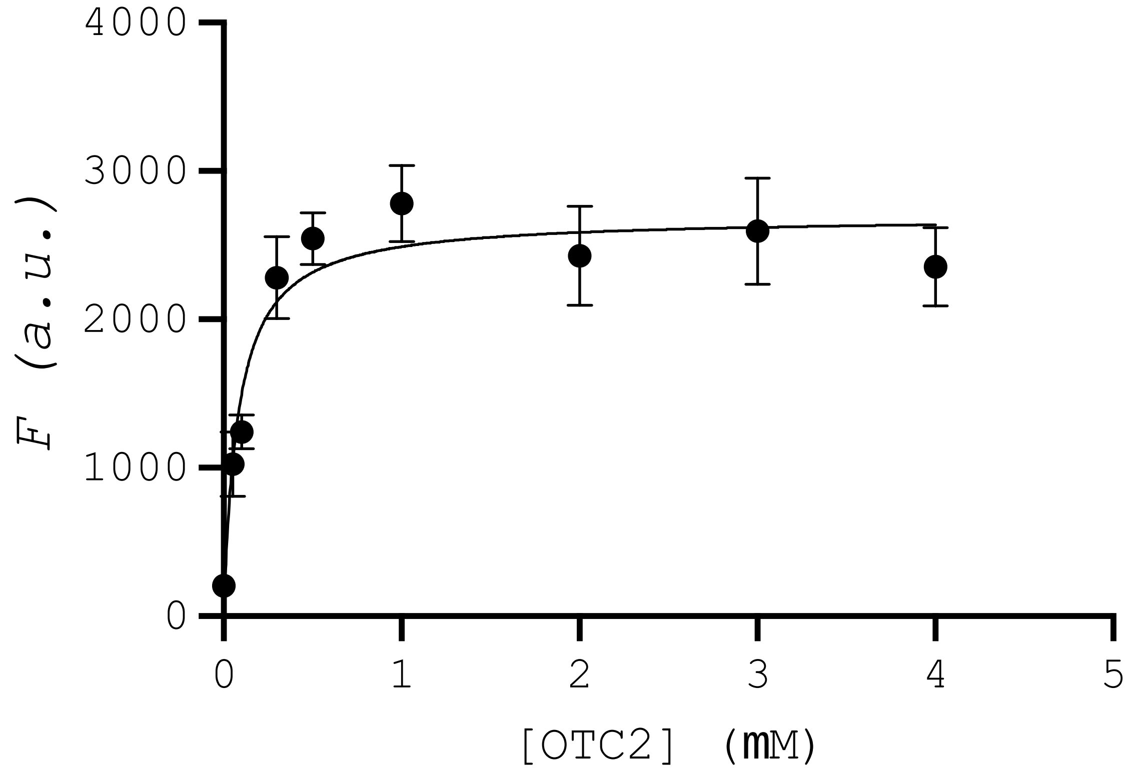

Figure S1. Titration curve of OTC2 into 100 nM OTC in 10 mM MES pH 6.0 buffer with 50 mM NaCl and 2 mM $MgCl_2$. The aptamer was allowed to bind for 12 minutes after each addition before reading. A fitted K_d of 0.081 μM was obtained.
